# Healthfulness of Woolens

**Published:** 1875-01

**Authors:** P. H. Hayes


### HEALTHFULNESS OF WOOLENS.

P. H. HAYES, M. D.

It is told of England's greatest admiral, Lord Nelson, that on one occasion, when he was about to put to sea, he discovered that the flannel undershirts ordered for his men had all been made six inches too short. He promptly refused to leave port until flannels of the right length were furnished. He was ridiculed and called an old granny. Yet the result was that while other crews of the fleet not thus flanneled were decimated by disease, Lord Nelson lost not a man, and the efficiency of his crew, as might be inferred, was as good as any two of the other.

Bonaparte said a great army moves on its stomach, yet for a whole soldier add Lord Nelson's flannels. In our own last national conflict the value of flannels for the health and comfort of a soldier was well understood, and now everywhere, among ordinary laborers and miners, the wearing of flannels is nearly, if not quite, universal. In the far west a fabric for this very purpose is found in all the markets, and known as "Miner's Flannel." McGregor, the English traveler, who explored the river Jordan in the Rob Roy, says of his outfit for dress, flannel always and everywhere, and all flannel. Physiology and experience, demonstrate that in order to the highest health, no other than wool textures should ever touch the skin.

The gist of comfort in clothing is that it shall retain a layer of warm air next the skin, not absolutely as rubber clothing would do, but so as to allow only a slow change in this air. This, woolen garments next the skin accomplish most effectually, as from their quality and texture heat and cold cannot pass quickly through them. This layer of warm air next the person, is itself also a non-conductor of heat and cold, and in its being kept from sudden change, lies the secret of health and comfort in dress. The under-woolens should therefore fit *loosely*, that there may be a large volume of warm air surrounding the person.

The most ordinary perspirations with other than woolens next the skin, are dangerous, for cotton and linen absorb and retain moisture in the very substance of their fiber, and then cling to the skin, so that not a particle of warm air can exist next the person, and every time one stirs a fresh chill creeps over the frame.

Flannel garments, on the contrary, *transport* moisture through their texture. This is because wool, being an animal fiber, is as it is growing penetrated with *sebum*, the oil of the skin of the sheep, and this oil prevents the filament of wool from becoming *wet* in a strict sense at all. Moisture passes *through* wool textures, glides between and over wool fibers, as it runs off from a duck's back, and for essentially the same reason. Blanket a sweating horse and return in half an hour and you will find the blanket covered with moisture like dew on the grass, on the outside, while the under surface is dry and your horse is also dry and warm. Reflect ! the warmth of the body of the horse expelled the moisture *through* the blanket. Cotton and linen become wet in the whole substance of their fabric, cling to the skin and evaporate from their whole substance. Notice then these wonderful qualities of woolen garments, that while they keep out the outer cold and damp, and keep in our layer of warm air, they also conduct through their substance and convey away promptly all moisture next the skin.

All the powers and actions of our bodies create vital warmth, and are in turn kept in vigorous health by this self-created warmth. All diseases impair the powers and actions of life, and by so much impair the power of the system to defend itself against cold and damp, and sudden atmospheric changes. In all affections of the rheumatic type, to which belong also the neuralgias and the gout, chilliness greatly aggravates the disease, and in acute rheumatism adds fearfully to the risk of heart disease. In Dr. Chambers' lectures on acute rheumatism, delivered in St. Mary's hospital, London, he says it is the standing order that no linen shall touch the skin, even a linen front to the shirt is dangerous. The body is kept wrapped up in blankets, and the newest and "*fluffiest*" that can be got are used. Even in health, next to running and jumping, nothing gives the heart so much hard work to do in circulating the blood as sudden changes from heat to cold. In rheumatic diseases, the great danger is that the rheumatic inflammation may attack the heart, but Dr. Chambers says he has never known a case of this transfer of inflammation to the heart, where the flannel treatment was began in time and rigidly enforced.

In all forms of invalidism, the first and most imperative necessity, is to aid the weak circulation of the blood, and promote vital warmth. The very texture of woolens keeps up a moderate excitation of the skin, which helps to fill it with blood warmth and magnetism, and thus creates, in part, what it so happily retains and saves. With nearly all invalids, not only is the circulation weak in the skin, but it is especially so in the feet. But if the feet be left cold, there is always a tendency to congestions of more vital organs with a general feeling of chilliness and *malaise*, and these arise in part from the increased burden of the heart in carrying on an impeded circulation. The amount of disease which creeps into the body through damp or cold feet is something frightful, if the facts were put together, and yet it is very often found that people who know well the value of flannel under-wear, have strangely left their feet out in the cold comfort of cotton stockings.

Cotton is no longer on the throne. Wool alone is imperial. Silk belongs to the outside aristocracy of the textiles. It is as much inferior to wool in the scale of health and comfort, as the worm that spins the thread is lower in the scale of being, than the animal of the snowy fleece. The delicate tissues of wool convey to the wearer a sense of comfort possessed by no other texture, and this instinctive sense of comfort means health.

Mothers! put your babies from neck to toe, and from the hour of their birth, into the softest woolens for their inner garments; let them sleep in changes *light* and *loose*; do not put on too much outer clothing, and do not *par-boil* them in close rooms, heated by air-tight stoves, and when teething comes, they shall not have colic and cramps, nor croup from the east wind, nor dysentery, nor cholera infantum, when the hot days and chilly nights of late summer and early autumn come on.

Flannels should *never* be left off; change to lighter material in June, and back again to heavier in October. Strange as it may appear, flannels are as necessary to qualify the *intemperies* of heat as of cold. It is literally true that we catch heat in the same sense that we catch cold. Colds produce congestions, so do heats. Witness sun-strokes, headaches from heat, congestions of liver and bowels, of which summer complaints are both the proof and the relief.

Sudden impressions of cold, when we are heated by exercise, or when we go out from a hot and close room, are alike liable to cause the *febricula*—the *diminutive fever*—which we call "a cold." Diminutive fever it usually is, yet right here began death for Washington, for Franklin, for Irving, for President Harrison, and what shall I say more? for does not every physician hear as an introduction to his patient's story, "I caught cold."

The skin carries off a third of all the impurities of the system. If its action is suddenly checked by cold, or depressed by heat, these impurities remain to poison the blood, induce congestion, and lay the foundation for fevers. Flannels promote the purifying work of the skin and render it uniform. Beware of very thick tight flannels, or those which become thick, matted and hard by ordinary washing, which *fulls* or *felts* the garment, and causes it to shrink in every direction and become heavy and impervious. Select light textures and be sure of a loose fit.

What is the mystery of this *fulling* or *felting* properly peculiar to wool? Every separate fiber is covered with little serratures pointing from the base of the fiber to its tip. These serratures are too delicate to be seen with the the naked eye. The microscope shows that when a piece of flannel is wet and subjected to hard pressure as in pounding or rubbing on the wash-board, the wool fibers begin a progressive movement in the thread both of the warp and the weft, and this movement continues until each fiber interlocks its serratures firmly with other fibers, but the point of any separate fiber is always directed toward the base of the one with which it interlocks.—Thus every wool filament seeks another with which it can interlock, and to which it adheres permanently, and the garment of wool every time it is pounded or rubbed grows shorter and thicker in every direction. A very intelligent lady tells me that she keeps the flannel for her family away from the wash-board and wringer, and washes them only as every lady has her laces washed, and she has no trouble from their fulling.

